# Cancer immune escape: the role of antigen presentation machinery

**DOI:** 10.1007/s00432-023-04737-8

**Published:** 2023-04-09

**Authors:** Anoop Kallingal, Mateusz Olszewski, Natalia Maciejewska, Wioletta Brankiewicz, Maciej Baginski

**Affiliations:** 1 Department of Pharmaceutical Technology and Biochemistry, Faculty of Chemistry, Gdansk University of Technology, Narutowicza St 11/12, 80-233 Gdansk, Poland; 2 Department of Medical Genetics, Institute of Clinical Medicine, University of Oslo, Oslo, Norway

**Keywords:** MHC-I, Epigenetic modulation, Immunopeptidome, Neoantigens

## Abstract

The mechanisms of antigen processing and presentation play a crucial role in the recognition and targeting of cancer cells by the immune system. Cancer cells can evade the immune system by downregulating or losing the expression of the proteins recognized by the immune cells as antigens, creating an immunosuppressive microenvironment, and altering their ability to process and present antigens. This review focuses on the mechanisms of cancer immune evasion with a specific emphasis on the role of antigen presentation machinery. The study of the immunopeptidome, or peptidomics, has provided insights into the mechanisms of cancer immune evasion and has potential applications in cancer diagnosis and treatment. Additionally, manipulating the epigenetic landscape of cancer cells plays a critical role in suppressing the immune response against cancer. Targeting these mechanisms through the use of HDACis, DNMTis, and combination therapies has the potential to improve the efficacy of cancer immunotherapy. However, further research is needed to fully understand the mechanisms of action and optimal use of these therapies in the clinical setting.

## Introduction

The role of antigen presentation in cancer immune cell escape is a complex and multifaceted topic that has been the subject of much research in recent years. Antigen presentation is the process by which cells in the immune system display foreign molecules, such as those from pathogens or cancer cells, on their surface for recognition by other immune cells (Zitvogel and Kroemer 2018). In the context of cancer, antigen presentation plays a crucial role in the ability of the immune system to identify and target cancer cells. However, cancer cells can evade the immune system by various mechanisms, including downregulating or losing the expression of the proteins recognized by the immune cells as antigens, a process known as an immune escape (Beatty and Gladney 2015). The process of antigen presentation begins with the cancer cells expressing proteins on their surface, which are then recognized by specialized immune cells called antigen-presenting cells (APCs) (Mpakali and Stratikos 2021). These APCs, such as dendritic cells, then internalize the cancer cell proteins and degrade them into smaller peptides. These peptides are then displayed on the surface of the APC, along with particular proteins called major histocompatibility complex (MHC) molecules (Blum et al. 2013). The MHC molecules act as a bridge between the cancer cell proteins and the immune cells responsible for recognizing and attacking cancer cells, called T cells. The T cells have T cell receptors (TCRs) that can recognize the cancer cell proteins displayed on the MHC molecules (Alberts et al. 2002). When a T cell recognizes a cancer cell protein displayed on an APC, it becomes activated and begins to divide and differentiate into specialized cells that can attack and destroy the cancer cells (Messerschmidt et al. 2016). Cancer cells can evade the immune system by downregulating or losing the expression of the proteins recognized by the immune cells as antigens (Beatty and Gladney 2015). This can happen by mutations in the cancer cells that affect the expression of these proteins or by the cancer cells creating an immunosuppressive microenvironment that prevents the immune cells from recognizing and attacking the cancer cells (Brody 2016). Some cancer cells can produce molecules called immune checkpoint inhibitors that bind to and inhibit the activity of T cells, preventing them from recognizing and attacking cancer cells (Lao et al. 2022).

Additionally, cancer cells can recruit immune cells that promote immune suppression, such as regulatory T cells and myeloid-derived suppressor cells, which further dampen the immune response against cancer (Brody 2016). Cancer cells can also evade the immune system by changing the location of the antigens within the cell, called the abscopal effect, where the cancer cells move the antigens to the inside of the cell, making them invisible to the immune system (Beatty and Gladney 2015; Alfonso et al. 2020). Recent research has shown that targeting the mechanisms of antigen presentation and immune escape can be an effective strategy for treating cancer. For example, drugs that block immune checkpoint inhibitors, such as anti-CTLA-4 and anti-PD-1/PD-L1, have been approved for use in several types of cancer and have shown promising results in clinical trials (Seidel et al. 2018; Rotte 2019). In a snapshot, antigen presentation plays a crucial role in the ability of the immune system to identify and target cancer cells. Understanding the mechanisms of antigen presentation and immune escape is crucial for developing effective cancer immunotherapies.

### Immune system and cancer

The immune system plays a crucial role in the development and progression of cancer (Gonzalez et al. 2018). Cancer cells develop from normal cells and can evade the immune system through various mechanisms; one of them is a process known as an immune escape. The immune system can recognize and target cancer cells through immunosurveillance. This process involves specialized immune cells, such as T cells and natural killer cells, that can detect and destroy cancer cells (Marcus et al. 2014; Gonzalez et al. 2018). The immune system also plays a role in shaping the microenvironment of the tumour. Tumour-associated macrophages, dendritic cells, and Treg cells are some of the cells found in the tumour microenvironment and play a role in cancer progression (Anderson and Simon 2020). Tumour-associated macrophages and dendritic cells can promote cancer cell growth by secreting factors that promote angiogenesis and inhibiting T cell activity. On the other hand Treg cells can suppress the immune response against cancer by inhibiting the activation and proliferation of T cells (Baay et al. 2011).

Another important mechanism in cancer progression is the ability of cancer cells to evade the immune system by downregulating or losing the expression of the proteins recognized by the immune cells as antigens (Dhatchinamoorthy et al. 2021). Recent research has shown that targeting the mechanisms of antigen presentation and immune escape can be an effective strategy for treating cancer. For example, drugs that block immune checkpoint inhibitors, such as anti-CTLA-4 and anti-PD-1/PD-L1, have been approved for use in several types of cancer and have shown promising results in clinical trials (Wojtukiewicz et al. 2021; Xiang et al. 2022; Sové et al. 2022). The immune system plays a crucial role in the development and progression of cancer. Understanding the mechanisms of immunosurveillance, immune escape, and the immune system's role in shaping the tumour microenvironment is crucial for developing effective cancer immunotherapies. Immune-based therapies, such as cancer vaccines and checkpoint inhibitors, have shown great promise in treating cancer and are expected to play a significant role in cancer treatment.

### Immune checkpoints and immune evasion in cancer

Cancer immune evasion refers to the ability of cancer cells to evade detection and destruction by the immune system (Vinay et al. 2015). This complex process involves multiple mechanisms that enable cancer cells to evade the immunosurveillance mechanisms of the body (Messerschmidt et al. 2016).

**Fig. 1:**
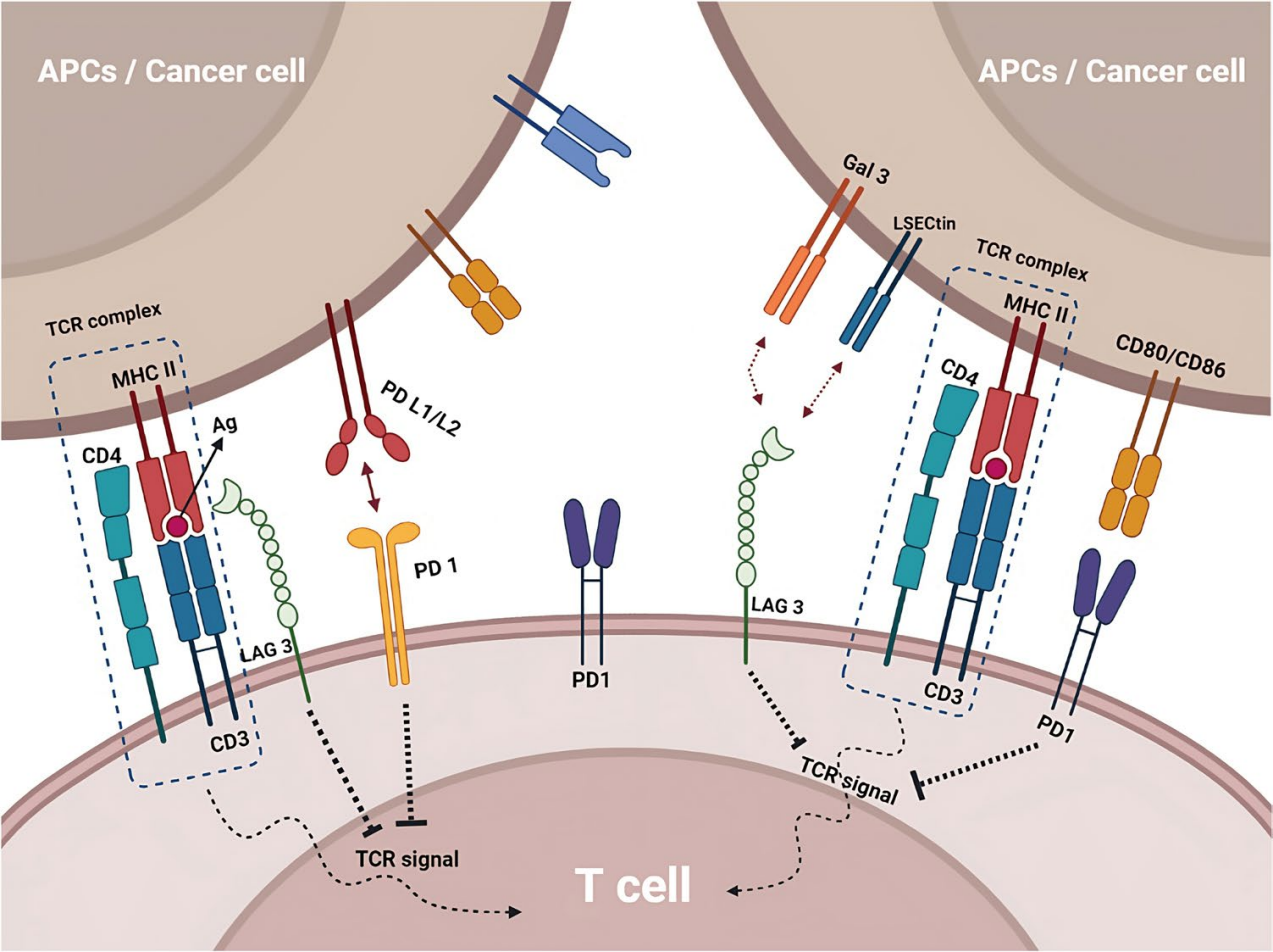
Immune checkpoint inhibitors, such as anti-CTLA-4 and anti-PD-1/PD-L1 drugs, enhance the immune response against cancer by blocking immune checkpoint pathways. Other checkpoint pathways, such as LAG-3 and TIGIT, are being investigated as potential targets for cancer therapy and may have synergistic effects when combined with other checkpoint inhibitors

Immune checkpoints are molecules or pathways that regulate the activation and function of the immune system. Immune checkpoint inhibitors are a class of drugs that block the function of these checkpoints, thereby enhancing the immune response against cancer cells (He and Xu 2020). One of the most well-known immune checkpoint pathways is the CTLA-4 pathway (Buchbinder and Desai 2016). CTLA-4 is a protein expressed on the surface of T cells that acts as an inhibitory receptor, blocking the activation and proliferation of T cells (Parry et al. 2005). Anti-CTLA-4 therapies, such as ipilimumab, act by binding to and blocking the function of CTLA-4, thereby enhancing the immune response against cancer cells (Callahan et al. 2010). Another critical immune checkpoint pathway is the PD-1/PD-L1. PD-1 is a protein expressed on the surface of T cells that interacts with PD-L1, which is expressed on the surface of cancer cells. This interaction blocks the activation and proliferation of T cells, allowing cancer cells to evade the immune response (Han et al. 2020). Anti-PD-1/PD-L1 treatments, such as nivolumab and pembrolizumab, work by binding to and inhibiting the interaction of PD-1 and PD-L1, increasing the immune response against cancer cells (Fessas et al. 2017) (Fig. 1).

Other immune checkpoint pathways, such as LAG-3 and TIGIT, are also being investigated as potential targets for cancer therapy. LAG-3 (lymphocyte activation gene 3) is a protein that binds to MHC class II molecules and regulates T cell activation and exhaustion (Ge et al. 2021; Huo et al. 2022). TIGIT (T cell immunoreceptor with Ig and ITIM domains) is a protein that binds to both T cells and immune cells and regulates T cell activation and function. Preclinical research has demonstrated a significant impact of these pathways, and clinical trials are currently being conducted to explore their potential as therapeutic cancer targets (Yue et al. 2022). LAG-3 and TIGIT have a unique mechanism of action compared to other immune checkpoint inhibitors, such as PD-1 and CTLA-4, and may have a synergistic effect when combined with these drugs. This could potentially lead to improved efficacy and reduced side effects. In preclinical studies, TIGIT and LAG-3 inhibitors are effective in combination with PD-1 inhibitors in various cancer models, such as melanoma, lung cancer, and ovarian cancer (De Sousa et al. 2018; Seidel et al. 2018; Willsmore et al. 2021).

### Antigen presentation in cancer

Antigen processing and presentation are crucial mechanisms by which the immune system recognizes and targets cancer cells. This process involves the recognition of cancer cell-associated antigens by APCs and their subsequent presentation on the surface of these cells in a form that can be recognized by T cells (Mpakali and Stratikos 2021). The antigen processing and presentation process begins with the internalization of cancer cell-associated antigens by APCs (Blum et al. 2013; Lee et al. 2020). Once inside the cell, the antigens are degraded into small peptides by a complex of enzymes called the proteasome. These peptides are then transported to the endoplasmic reticulum, complex with MHC molecules (Rock et al. 2010). MHC molecules are specialized proteins that are essential for the recognition of antigens by T cells. There are two main types of MHC molecules: MHC class I and MHC class II. MHC class I molecules are expressed on the surface of all nucleated cells, including cancer cells, and present peptides derived from intracellular antigens. On the other hand, MHC class II molecules are expressed primarily on the surface of APCs and present peptides derived from extracellular antigens (Wieczorek et al. 2017).

**Fig. 2:**
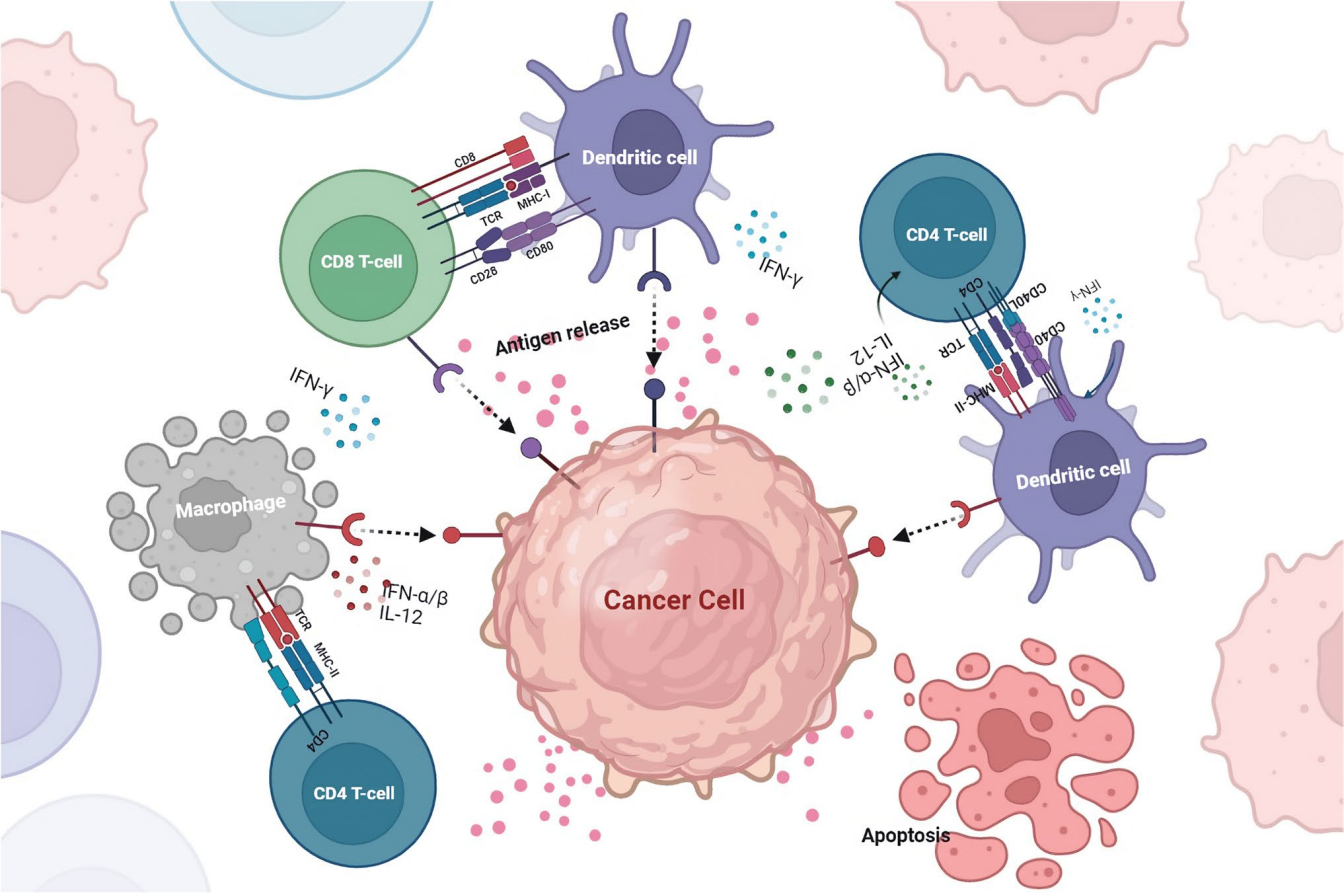
APCs internalize cancer cell-associated antigens and degrade them into small peptides, which are then presented on the surface of APCs as MHC-peptide complexes that can be recognized by T cells. Cancer cells can evade the immune system by downregulating or losing the expression of antigen proteins, altering their ability to process and present antigens, or creating an immunosuppressive microenvironment

The MHC-peptide complex is then transported to the cell surface, where it can be recognized by T cells. T cells have specialized T cell receptors (TCRs) that recognize the MHC-peptide complex (Alberts et al. 2002). When a T cell recognizes a cancer cell-associated antigen displayed on an APC, it becomes activated and begins to divide and differentiate into specialized cells that can attack and destroy the cancer cells (Kunimasa and Goto 2020). However, cancer cells can evade the immune system by downregulating or losing the expression of the proteins recognized by the immune cells as antigens. This can happen by mutations in the cancer cells that affect the expression of these proteins or by the cancer cells creating an immunosuppressive microenvironment that prevents the immune cells from recognizing and attacking the cancer cells (Beatty and Gladney 2015) (Fig. 2). Many reports have shown that cancer cells can also evade the immune system by altering their ability to process and present antigens. For example, some cancer cells can downregulate the expression of MHC molecules, making them invisible to the immune system (Mittal et al. 2014; Reeves and James 2017; Kulkarni et al. 2019). Cancer cells can also interfere with the activity of the proteasome, thereby preventing the degradation of cancer cell-associated antigens (Mittal et al. 2014; Reeves and James 2017; Kulkarni et al. 2019).

### MHC 1 in antigen presentation

Major histocompatibility complex class I (MHC-I) molecules play a critical role in antigen presentation. These molecules are expressed on the surface of all nucleated cells, including cancer cells, and are responsible for the presentation of peptides derived from intracellular antigens to CD8 + T cells, also known as cytotoxic T cells (van den Elsen 2011; Wang et al. 2019). The MHC-I molecule comprises two main components: the heavy chain, encoded by the HLA gene, and the beta-2-microglobulin (β2m), a non-polymorphic component. The heavy chain comprises three main domains: the α1, α2, and α3. The α1 and α2 domains bind the MHC-I molecule to the peptide, while the α3 domain is responsible for interacting with the CD8 T-cell receptor (Cruz-Tapias et al. 2013). The process of MHC-I presentation begins with the internalization of antigens by the cell. Once an antigen enters a cell, a group of enzymes called the proteasome breaks it down into a little peptide.

**Fig. 3:**
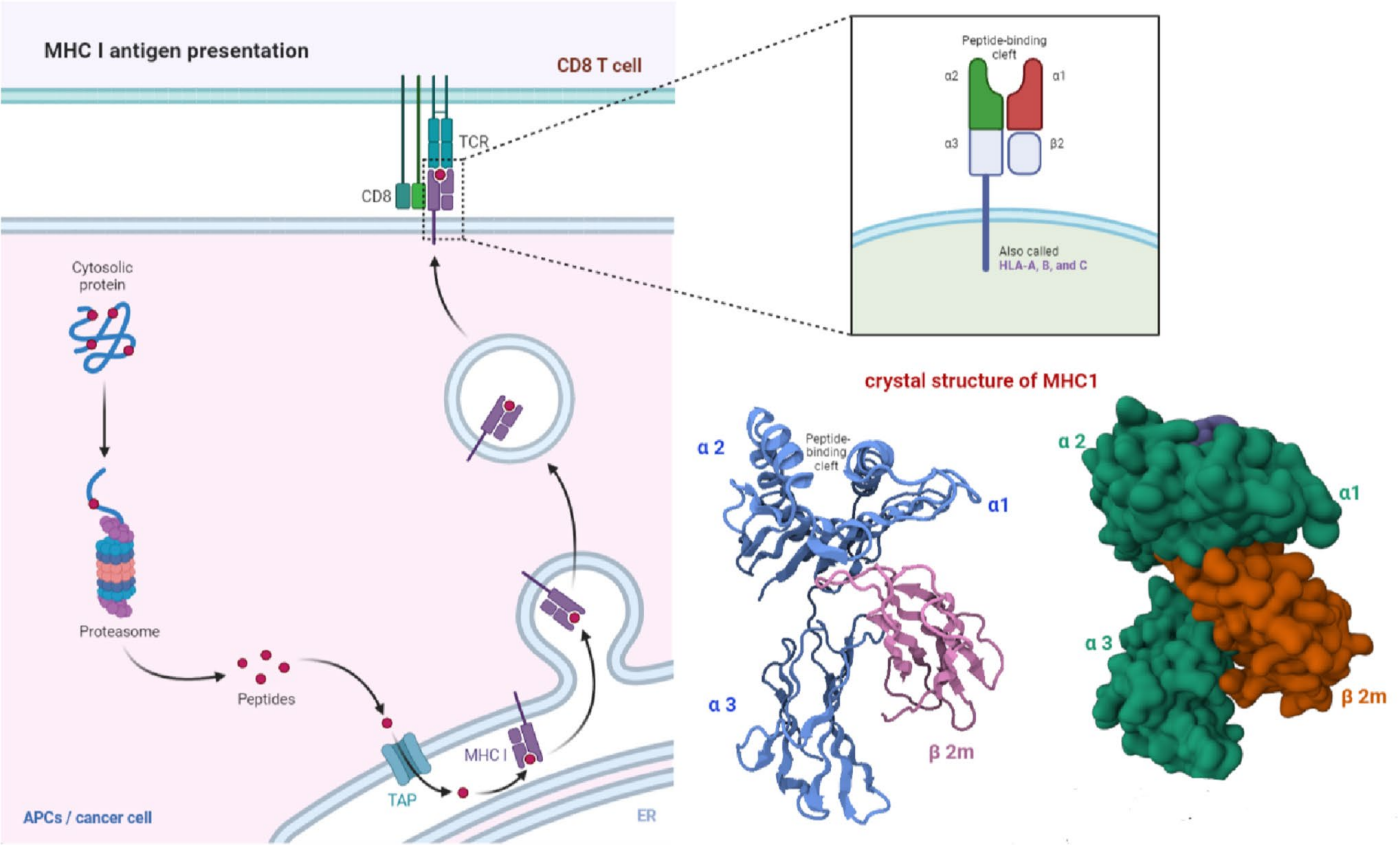
MHC-I antigen presentation. MHC-I molecules on the cell surface present intracellular antigen peptides to CD8 + T cells. Cancer cells can evade the immune system by downregulating antigen expression or altering antigen processing and presentation on MHC-I

Peptide loading delivers these peptides to the endoplasmic reticulum, where they interact with the MHC-I molecule. The MHC-I-peptide complex is then transported to the cell surface, where it can be recognized by CD8 + T cells (Hewitt 2003). The binding of the peptide to the MHC-I molecule is mediated by the peptide-binding groove, which is composed of the α1 and α2 domains. The peptide-binding groove can only bind to peptides that are 8–10 amino acids long. Once the peptide is bound to the MHC-I molecule, it is transported to the cell surface (Fig. 3) (Zacharias and Springer 2004). Downregulating or removing proteins that express antigens allows cancer cells to evade the immune system. The ability of cancer cells to process and present antigens on MHC-I molecules can change if they develop an immunosuppressive microenvironment or experience protein expression mutations. Understanding the mechanisms of MHC-I presentation in cancer is crucial for developing effective cancer immunotherapies.

### Immunopeptidome and cancer

The immunopeptidome is the set of peptides presented by MHC molecules on the surface of cells (Yewdell 2022a). These peptides are derived from the degradation of intracellular proteins and are essential for recognizing cancer cells by the immune system. The study of the immunopeptidome, also known as peptidomics, has revealed insights into the mechanisms of cancer immune evasion and has potential applications in cancer diagnosis and treatment (Synowsky et al. 2017; Yewdell 2022b). One of the critical roles of the immunopeptidome in cancer is its ability to identify unique peptides specific to cancer cells. These cancer-specific peptides, also known as neoantigens, can be used to develop personalized cancer vaccines targeting the unique mutations in an individual's cancer. Neoantigen-based vaccines have shown promising results in clinical trials and are expected to play an essential role in the future of cancer immunotherapy (D’Amico et al. 2022; Ouspenskaia et al. 2022). Another essential role of the immunopeptidome in cancer is its ability to provide insights into the mechanisms of cancer immune evasion. The study of the immunopeptidome can reveal which proteins are being presented by MHC molecules and which are not, providing insight into the mechanisms of cancer immune evasion (León-Letelier et al. 2022). The immunopeptidome can also provide valuable information for cancer diagnosis, such as immunopeptidome-based cancer diagnostics, tumour-associated antigen (TAA) testing, MHC class I tetramer staining and mass spectrometry-based peptidomics. Additionally, the study of the immunopeptidome can provide insights into the progression of cancer and the response to treatment by monitoring changes in the peptides presented by MHC molecules (Dersh et al. 2021).

### Tumor antigen expression, presentation and control

**Fig. 4:**
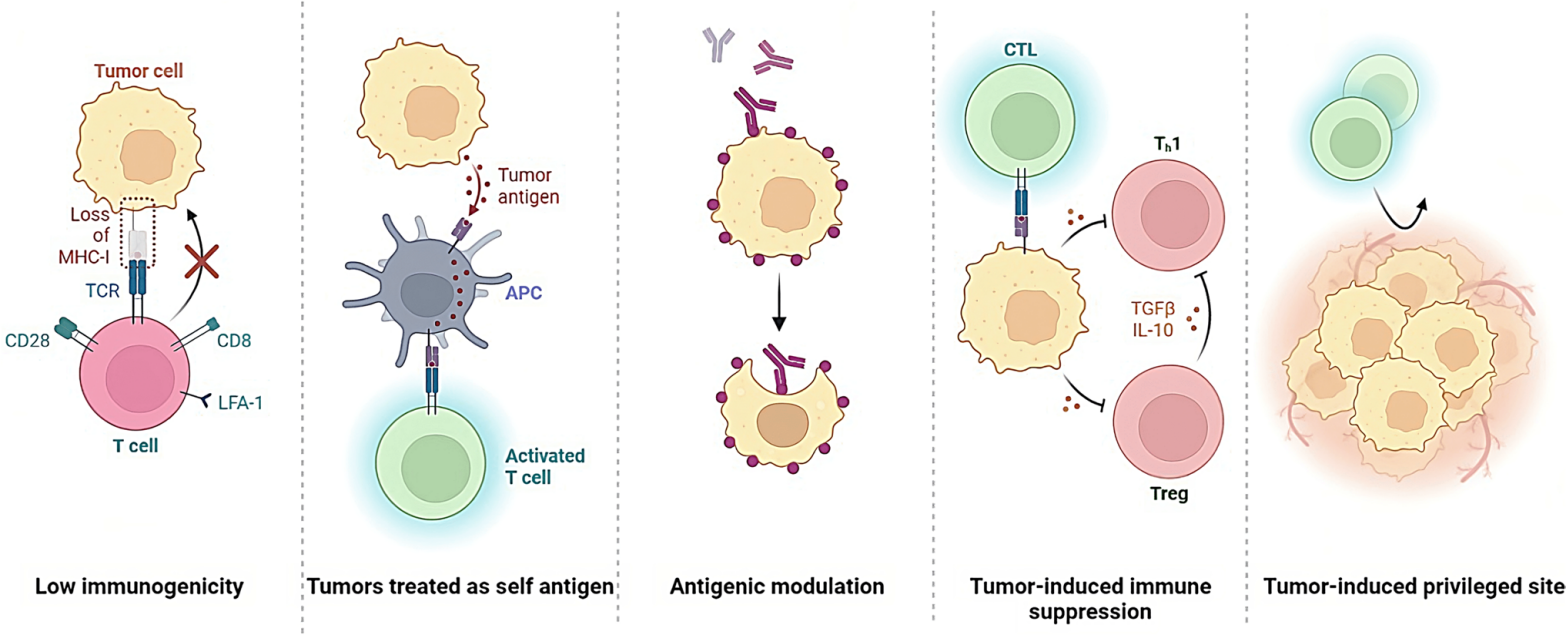
Tumors can evade detection and destruction by the immune system, thereby allowing for uncontrolled growth and progression. This process is referred to as immune evasion and is a complex mechanism that involves the downregulation or loss of antigens recognized by immune cells, the creation of an immunosuppressive microenvironment, and interaction with immune checkpoint pathways

The control of tumour antigen expression and presentation is a critical aspect of cancer biology that significantly impacts the immune system's ability to recognize and target cancer cells (Whiteside 2006). Tumours evade immune recognition through various mechanisms, such as the downregulation of antigens recognized by immune cells, the creation of an immunosuppressive microenvironment, and interaction with immune checkpoint pathways. Tumour antigens are molecules expressed on the surface of cancer cells and recognized by the immune system as foreign (Fig. 4).

Cancer cells can regulate tumour antigen expression via epigenetics, like DNA structure changes (methylation, histone modification). They can also reduce antigen expression, hide from the immune system, and inhibit antigen-presenting cells/T cells (TGF-beta, IL-10) from suppressing immune response.(Gibney and Nolan 2010). Another mechanism by which cancer cells can control the expression of tumour antigens is through the manipulation of the proteasome and the MHC molecules (Boulpicante et al. 2020). The proteasome is a complex of enzymes responsible for degrading intracellular proteins, including tumour antigens, into peptides that MHC molecules can present. Cancer cells can interfere with the activity of the proteasome, thereby preventing the degradation of cancer cell-associated antigens and avoiding the presentation of the antigens on the MHC molecules (Chen et al. 2022). Cancer cells can also downregulate the expression of MHC molecules, thus making them invisible to the immune system and avoiding antigen presentation, or manipulate the structure of the MHC molecules, such as altering the peptide binding affinity, which can prevent the presentation of the cancer-associated antigens (Hewitt 2003; Rock et al. 2010; Blum et al. 2013).

### Epigenetic modulation of immunotherapy

One mechanism by which cancer cells can control the expression of tumour antigens is through epigenetic regulation. Epigenetics refers to the regulation of gene expression through changes in the structure of DNA, such as methylation and histone modification, rather than changes in the genetic code itself (Gibney and Nolan 2010). Cancer cells can alter the epigenetic landscape to downregulate the expression of tumour antigens, making them invisible to the immune system. Cancer cells can also secrete factors that inhibit the activity of antigen-presenting cells and T cells, such as TGF-beta and IL-10, which further suppress the immune response (Thepmalee et al. 2018). Epigenetic modulation of antitumor immunity has been an active area of research in recent years and has been found to have potential applications in cancer immunotherapy (Gibney and Nolan 2010). Cancer cells' manipulation of the epigenetic landscape has been shown to play a critical role in suppressing the immune response against cancer. By targeting these mechanisms, it is possible to improve the efficacy of cancer immunotherapy (Liu et al. 2022a). One way in which epigenetic modulation can be targeted is through the use of histone deacetylase inhibitors (HDACis). HDACis are a class of drugs that inhibit the activity of histone deacetylases, enzymes that remove acetyl groups from histones, leading to the repression of gene expression. HDACis have been shown to enhance the maturation of dendritic cells and increase the presentation of tumour antigens, thus enhancing the immune response against cancer (Gryder et al. 2012).

**Fig. 5:**
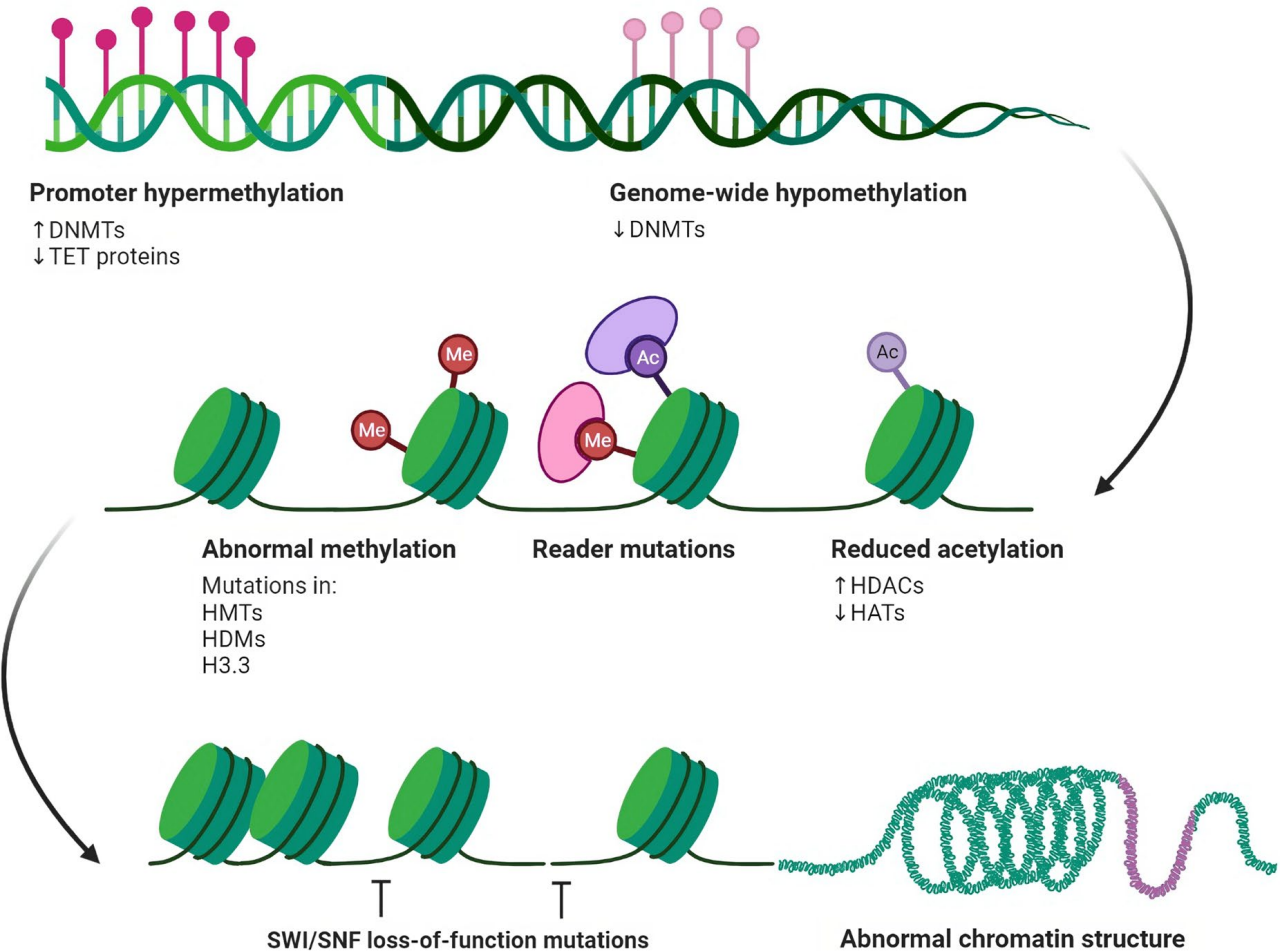
Diagram illustrating the epigenetic regulation of chromatin accessibility and gene expression in cells. Nucleosomes, formed by DNA wrapped around histone octamers, are depicted as blue cylinders. Epigenetic modifications are depicted as dynamic interactions between chromatin components and enzymes, including histone methylation/demethylation, histone acetylation/deacetylation, and DNA methylation. Chromatin remodelling also plays a role in regulating gene expression

Another way to target epigenetic modulation is through DNA methyltransferase inhibitors (DNMTis) (Hu et al. 2021). DNMTis are a class of drugs that inhibit the activity of DNA methyltransferases, enzymes that add methyl groups to DNA, leading to the repression of gene expression. DNMTis have been shown to increase the expression of genes involved in the immune response, such as MHC molecules, and modulate the expression of genes involved in immune evasions, such as PD-L1 (Dan et al. 2019) (Fig. 5). The combination therapies that combine epigenetic modulation with other immunotherapeutic strategies, such as checkpoint inhibitors, have also yielded promising results in clinical trials. For example, combining HDACis with PD-1/PD-L1 inhibitors has enhanced the response to treatment in multiple cancer types (Mazzone et al. 2017; Liu et al. 2022b).

It is important to note that while the use of these epigenetic modulation therapies has shown promising results in preclinical and clinical studies, more research is needed to fully understand the mechanisms of action and optimal use in the clinical setting. Further research is also needed to understand these therapies' potential side effects and long-term safety.

### Antigen presentation machinery components, modulation and their defects

The antigen processing machinery (APM) plays a critical role in developing an effective antitumor immune response (Maggs et al. 2021). The APM is a group of cellular structures and molecules responsible for processing and presenting APCs to T cells. Defects in the APM can compromise the ability of the immune system to recognize and respond to cancer cells, leading to the development of tumours that evade destruction by the immune system (Mpakali and Stratikos 2021). The major components of the APM include proteasomes, which are responsible for the degradation of proteins into peptides; TAP (transporter associated with antigen processing), which transports the peptides from the cytosol to the endoplasmic reticulum (ER); and MHC (major histocompatibility complex) molecules, which present the peptides on the surface of APCs to T cells. A growing body of evidence suggests that defects in the APM can contribute to cancer development. For example, mutations in the genes encoding the proteasomes or TAP can reduce the ability to generate peptides that can be presented on MHC molecules (Reiman et al. 2007). This can limit the ability of the immune system to recognize and respond to cancer cells. Additionally, defects in MHC molecules can result in a decreased ability to mount an immune response against certain infections and cancer (Charles et al. 2001; Dassa 2003).

Cancer cells can modulate antigen presentation in several ways to evade recognition and destruction by the immune system. Cancer cells can do this by deregulation of MHC molecules; Cancer cells can reduce the expression of MHC molecules on their surface, making them less visible to T cells and harder to target. Disruption of antigen processing; Cancer cells can interfere with the normal processing of antigens within the cell, making it harder for APCs to present them on MHC molecules. Production of immunosuppressive molecules; Cancer cells can produce molecules that suppress the immune response, such as TGF-beta and IDO, making it harder for T cells to recognize and attack cancer cells. Recruitment of immune-suppressive cells; Cancer cells can recruit immune cells that suppress the immune response, such as Tregs and MDSCs, to the tumour microenvironment (Vinay et al. 2015; Parcesepe et al. 2016; Mergener and Peña-Llopis 2022).

Defects in any of these components can result in a compromised immune response. For example, mutations in MHC molecules can result in a condition called MHC deficiency, which leads to a decreased ability to mount an immune response against certain infections. Similarly, TCR defects can result in T cell dysfunction and increased susceptibility to infections. Defects in the antigen presentation machinery can significantly impact the immune system's ability to recognize and respond to cancer cells, and understanding these defects can inform the development of new immunotherapies for cancer (Mpakali and Stratikos 2021). The development of immunotherapies for cancer has been a promising approach to targeting tumours that evade destruction by the immune system. These therapies aim to re-activate the patient's immune system to recognize and attack cancer cells. This can include checkpoint inhibitors, which block the immune-suppressive signals emitted by cancer cells and allow T cells to recognize and attack the tumour, and CAR T-cell therapy, which genetically modifies a patient's T cells to recognize and attack cancer cells (Filley et al. 2018).

### Neoantigens in cancer immunotherapy

Neoantigens are a class of tumour-specific antigens generated by genetic mutations in cancer cells. They are not present in normal cells and, thus, represent a unique target for cancer immunotherapy. Identifying and characterising neoantigens have led to the development of new immunotherapeutic strategies for cancer treatment (Zhu and Liu 2021). The process of neoantigen identification begins with the sequencing of a patient's tumour and normal DNA (Zhu and Liu 2021). Algorithms are then used to identify potential neoantigens based on their predicted binding to MHC molecules and their potential to be presented on the cell surface. These potential neoantigens are further validated through functional assays, such as T-cell assays, to confirm their ability to elicit a T-cell response (Garcia-Garijo et al. 2019; Zaidi et al. 2020). Once identified, neoantigens can be used to develop personalized cancer vaccines (Blass and Ott 2021). These vaccines can target specific mutations in an individual's tumour and stimulate an immune response against cancer cells. The vaccines can be either ex vivo, where T cells are extracted from the patient, genetically modified to recognize the neoantigens, and then re-infused back into the patient or in vivo, where the patient is administered with the neoantigen peptides (Xie et al. 2023).

Recent clinical trials have demonstrated the safety and efficacy of personalized neoantigen cancer vaccines (Fritah et al. 2022). The results have shown that these vaccines can induce antitumor T-cell responses and result in durable clinical responses in a subset of patients with advanced cancer. Additionally, a combination of neoantigen vaccine with checkpoint inhibitors has shown to be more effective in inducing antitumor T-cell response and, in some cases, led to complete remission of the disease (Liao and Zhang 2021). Furthermore, the identification of neoantigens has also led to the development of neoantigen-targeting T-cell therapies, such as CAR-T cell therapy. In this approach, T cells are genetically modified to express a CAR specific for a neoantigen and then re-infused back into the patient. These therapies have shown effective in inducing long-lasting responses in patients with advanced cancer (Wang and Cao 2020).

## Conclusion

The antigen processing and presentation mechanisms play a critical role in the immune system's recognition and targeting of cancer cells. Cancer cells can avoid immune detection by downregulating or losing the expression of proteins recognised as antigens, creating an immunosuppressive microenvironment, and altering their ability to process and present antigens. The study of the immunopeptidome, or peptidomics, has provided insights into the mechanisms of cancer immune evasion and has potential applications in cancer diagnosis and treatment. One mechanism by which cancer cells can control the expression of tumour antigens is through epigenetic regulation, such as methylation and histone modification; cancer cells can alter the epigenetic landscape to downregulate the expression of tumour antigens, making them invisible to the immune system. Additionally, cancer cells can manipulate the microenvironment, interfere with the activity of the proteasome and MHC molecules, and downregulate the expression of MHC molecules to avoid the presentation of antigens. Recent advances in cancer genomics and molecular biology have allowed the identification of unique antigens present in cancer cells but not in normal cells, known as "neoantigens." These neoantigens can be used to develop cancer vaccines and CAR-T cell therapy that target the specific mutations present in an individual's tumour, leading to the re-activation of the patient's immune system to recognize and attack cancer cells. Targeting the epigenetic mechanisms that cancer cells use to evade the immune system can improve cancer immunotherapy, such as using HDACis, DNMTis, and combination therapies. However, it's important to note that more research is needed to fully understand the mechanisms of action and optimal use of these therapies in the clinical setting. In snapshot, controlling tumour antigen expression and presentation is a critical aspect of cancer biology that significantly impacts the immune system's ability to recognize and target cancer cells. Understanding these mechanisms is crucial for developing effective cancer immunotherapies that target the mechanisms of antigen expression and presentation in cancer cells and for a better understanding of the epigenetic modulation of antitumor immunity for improved cancer immunotherapy.

## Data Availability

All data generated or analysed during this study are included in this published article.
